# The Importance of Laser Beam Power on the Microstructure and Wear Behavior of Al-WC Composite Layers Produced by Laser Surface Alloying

**DOI:** 10.3390/ma18091899

**Published:** 2025-04-22

**Authors:** Natalia Makuch, Piotr Dziarski

**Affiliations:** Institute of Materials Science and Engineering, Poznan University of Technology, Pl. M. Sklodowskiej-Curie 5, 60-965 Poznan, Poland; piotr.dziarski@put.poznan.pl

**Keywords:** laser surface alloying, tungsten carbide, aluminum alloys, surface topography, wear resistance

## Abstract

Laser alloying was used to form metal matrix composite layers strengthened by WC particles. The process parameters were selected in such a way that there was no complete melting of the WC particles. Four different laser beam powers (from 0.65 kW to 1.3 kW) were used, generating different temperature distributions during processing. The temperature across the laser track axis was determined according to the mathematical model proposed by Ashby and Esterling. All layers produced contained unmelted WC particles in an aluminum-based matrix. The depth of the WC-Al composite layers strongly depended on the applied laser beam power. The lowest thickness of 198 ± 36 µm was measured for the layer produced at a laser beam power of 0.65 kW. A twofold increase in power *P* was the reason for obtaining a thickness *th_AZ_* = 387 ± 21 µm. The power of the laser beam also affected the percentage of the substrate material (7075 alloy) in the molten pool during the laser processing. As a result, the highest amount of substrate material was obtained for the WC-Al composite layer produced using the highest laser beam power *P* = 1.3 kW. Simultaneously, this layer was characterized by the lowest percentage of tungsten carbide particles in this layer. The temperature profile along the axis of the laser track and also the maximum temperature reached confirmed the difference in the bonding between the reinforcing WC particles and the metal matrix. For *P* = 0.65 kW, too low a temperature was reached for the tungsten carbide particles to overmelt, resulting in poor bonding to the metallic matrix in the layer. Moreover, the layer showed serious defects such as discontinuity, porosity, and cracks. As a result, the WC-Al composite layer produced at the lowest laser beam power was characterized by a wear resistance lower (*I_mw_* = 6.094 mg/cm^2^/h) than the 7075 alloy without surface layer (*I_mw_* = 5.288 mg/cm^2^). The highest wear resistance was characteristic of the 7075 alloy laser alloyed with a laser beam power equal to 1.17 kW (*I_mw_* = 2.475 mg/cm^2^/h). This layer showed satisfactory quality and adhesion to the substrate material.

## 1. Introduction

Aluminum and its alloys are low-density materials characterized by both high specific strength and high ductility. These characteristics are the reason for its wide industrial use in, for example, aerospace, transportation, and automotive applications [1]. Additive techniques based on the use of laser beams [2,3] are increasingly common methods of manufacturing aluminum alloys. In addition, aluminum and its alloys are often the matrix in reinforcing ceramic particle–metal matrix composites [4,5,6]. Laser techniques are also often used to increase the wear resistance of aluminum and its alloys [7,8]. Laser surface processing techniques are unique in their ability to produce a variety of surface layers on an unlimited number of metallic substrate materials [9,10]. Laser surface modification of aluminum alloys allows for the formation of alloyed layers enriched with metallic or non-metallic elements [11,12,13,14,15,16,17,18] and also for the production of composite layers containing strengthening particles in a metallic matrix [19,20,21,22,23,24,25,26,27,28].

In particular, the production of a metal–matrix composite containing hard ceramic particles appears to be a promising technique for increasing the wear resistance of an aluminum alloy. The most attractive ceramic particles used as a strengthening phase in metal-matrix composite layers are as follows: tungsten carbide WC [20], titanium carbide TiC [21,22,23,24,25], and silicon carbide SiC [26,27].

The formation of a laser alloyed layer on an AA6061 alloy by laser surface melting with Mo-WC powder was presented in this paper [20]. The influence of the molybdenum content in the alloying powder on the microstructure and properties was analyzed. The bonding strength between the tungsten carbide particles and the metal matrix was improved by the presence of dendrites on the partially melted boundary of the tungsten carbide. The formation of a metal–matrix composite layer containing WC particles provided a major improvement in wear resistance compared to the untreated AA6061 aluminum alloy. However, it was found that an increase in the amount of tungsten carbide particles in the layer could cause cracks to form in the formed layer [20]. In the paper [23], ceramic TiC particles were used in order to produce a composite layer of the ceramic-metal type on the surface of 6061 aluminum alloy. Such a layer was characterized by increased hardness and wear resistance compared to the alloy without laser surface modification. The advantageous effect of the presence of titanium carbides in the composite layer formed on the AA6061 alloy was analyzed in the paper [25]. A layer of a high thickness of 500 µm was produced. Simultaneously, the reinforcing TiC particles were fine-grained (<3 µm) and homogeneously distributed in the matrix. The layer produced increased the hardness ninefold (to a maximum of 650 HV) compared to the substrate material (75 HV). The other ceramic particles used as a strengthening phase in a metal–matrix composite layer were SiC [26]. It was proven that the production of metal–matrix composite layers reinforced by SiC particles improved the hardness (143–318 HV, depending on the composition of the alloying powder) compared to the hardness of the substrate material (24 HV). An improvement of 19–82% was achieved with respect to the wear resistance of the AA1200 alloy. The highest wear resistance was obtained when the percentage of SiC particles in the powder mixture used for laser alloying was the highest (40%).

The positive effects of using hard ceramic phases as a reinforcing phase in composite layers became the inspiration in this study to produce WC-Al layers on the 7075 alloy. Four different laser beam powers were used: 0.65 kW, 0.91 kW, 1.13 kW, and 1.3 kW. The influence of the applied power on the temperature distribution along the laser track axis, the quality of the produced WC-Al composite layers, and their wear resistance were analyzed. The results not only allowed us to analyze the effect of the power of the laser beam on the depth of the alloyed zone *th_AZ_*, but, most importantly, provided important information on the role of the molten pool temperature on the bonding of ceramic particles to the metallic matrix. Analyzing images of the topography of the worn surfaces proved the theory of poor bonding of tungsten carbides to the aluminum-based matrix when using a power of 0.65 kW. Usually, a lower laser beam power had an advantageous effect on wear resistance, as described in the work [29], due to the high percentage of reinforcing particles. However, in the case of composite layers containing materials that differ strongly in melting points (in this case by more than three times), this phenomenon was disrupted. The reasons for this situation have been extensively explained in this paper.

## 2. Materials and Methods

### 2.1. Materials

A 7075 aluminum alloy was used as the substrate material for this study. Its nominal chemical composition was as follows: 5.1–6.1 wt.% Zn, 1.2–2 wt.% Cu, 2.1–2.9 wt.% Mg, 0.18–0.28 wt.% Cr, max. 0.5 wt.% Fe, max. 0.4 wt.% Si, balance Al. This alloy is widely used, e.g., in the aerospace industry, due to an excellent compromise between its density and strength. The alloying material had a form of paste containing tungsten carbide (particle sizes of 25–50 µm) and aluminum powder (particle sizes of 5–10 µm). The alloying paste also contained polyvinyl alcohol as a binder. The samples were ring-shaped with the following dimensions: external diameter of 20 mm, internal diameter of 12 mm, height of 12 mm.

### 2.2. Laser Surface Alloying

**Figure 1:**
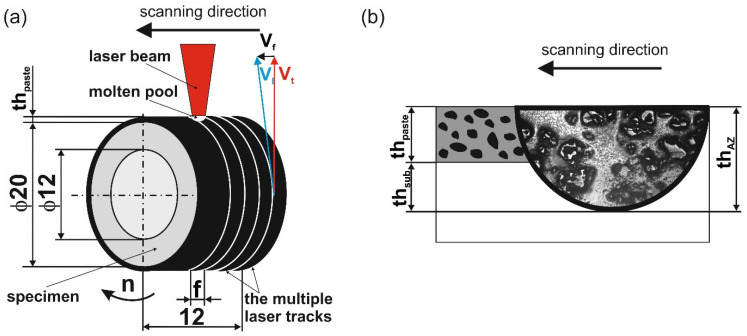
The scheme of laser alloying by re-melting technique (**a**) and details of alloyed zone formation (**b**): *v*_f_—feed rate; *v*_l_—scanning rate; *v*_t_— tangential speed; n—rotational speed; f—distance between the axes of adjacent tracks; *th_paste_*—thickness of pre-coated paste with alloying material; *th_sub_*—thickness of the re-melted substrate material; *th_AZ_*—thickness of produced laser surface alloyed zone.

The scheme of the laser surface modification process is shown in Figure 1a. Laser surface alloying has been arranged as a remelting method in which the treated material is pre-coated with an alloying material in the form of a paste, and then both materials (alloying and alloyed) are re-melted with a laser beam. The alloying paste was composed of WC and aluminum powders, as well as polyvinyl alcohol as a binder. The weight ratio of the powder was 70% WC and 30% Al.

Before the laser surface alloying process, the external cylindrical surface was pre-coated with a paste containing alloying material. The thickness of the paste, *th_paste,_* was 160 µm. The interaction between the laser beam and the treated surface increased the temperature and caused melting of the alloying paste of thickness *th_paste_* and some part of the substrate material at a depth of *th_sub_* (Figure 1b). The thickness of the re-melted substrate material *th_sub_* depends on laser alloying parameters: laser beam power *P*, laser beam radius *r_B_*, and scanning rate *v_l_*. The *th_sub_* value was calculated as the difference between the thickness of the alloyed zone *th_AZ_* (calculated from microstructure images) and the thickness of the pre-coated paste containing alloying powder *th_paste_*. The produced laser surface alloyed zone with the characteristic shape of the laser track can have a different thickness *th_AZ_*.

The WC-Al composite layers were produced using the CO_2_ molecular laser (TLF 2600 Turbo (TRUMPF, Poznan, Poland). All processes were conducted using a laser beam radius *r_B_* of 1 mm, scanning rate (*v_l_*) of 2.88 m/min, feed rate (*v_f_*) of 0.56 mm per revolution, and rotational speed (*n*) of 45.85 min^−1^. The only variable parameter during processing was the power of the laser beam, and it was 0.65 kW, 0.91 kW, 1.17 kW, and 1.3 kW. During laser surface treatment, the samples were protected from oxidation by an argon atmosphere. For this purpose, a protective chamber was used, which was filled with protective gas during laser processing. The WC-Al composite layers were arranged as multiple tracks, and the distance between the centers of adjacent tracks was 0.56 mm (Figure 1a). The aim of the laser surface alloying of the 7075 alloy was to produce WC-Al composite layers in which WC particles play an important role during wear. Therefore, the laser alloying parameters were selected so that the tungsten carbide particles were not completely melted.

### 2.3. Characterization of Produced WC-Al Composite Layers

Metallographic preparation by cutting, grinding, and polishing was used to prepare the cross-sectional samples. Microstructural details were observed using an optical microscope (OM) LAB-40 (OPTA-TECH, Poznan, Poland). The thicknesses of the WC-Al composite layers were determined as the average value from 100 measurements taken on OM images at equal intervals. The surface topography of the layers produced at laser beam powers of 0.65 kW and 1.17 kW was analyzed using a VHX7000 digital microscope (KEYENCE, Poznan, Poland). Two- and three-dimensional images of the surface were recorded.

**Figure 2:**
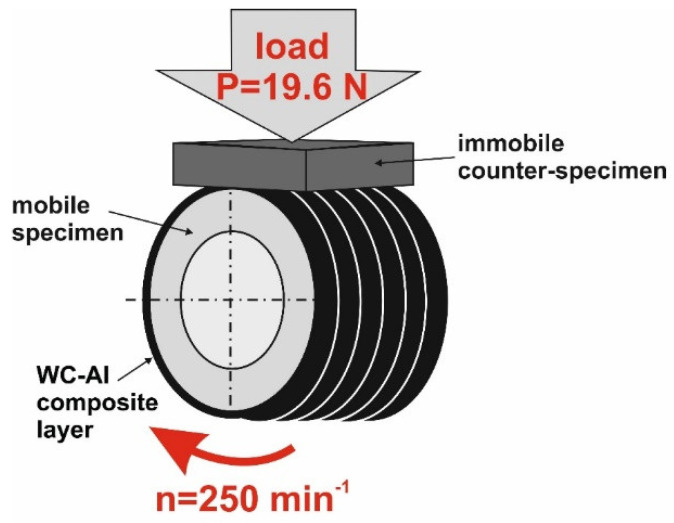
The scheme of the friction pair consisting of mobile specimen and immobile counter-specimen.

Tribological properties were tested using a friction pair presented in Figure 2. The block-on-ring test was applied. Although the surface roughness was changed after laser surface alloying, the surfaces of the specimens were not prepared before the wear test. During the test, the specimen rotated at a speed of n = 250 min^−1^, and the stationary counter-specimen was loaded from the top with a load of *P* = 19.6 N. The counter-specimen was plate-shaped S20S sintered carbide (BAILDONIT, Poznań, Poland) with dimensions: 12 × 12 × 5 mm. The counter-specimen was mounted in a holder, and the load used was uniformly distributed over the entire top surface of the counter-specimen. During the test, the position of the counter-specimen did not change, and the specimen was mounted in the same way relative to the counter-specimen after each weighing, which occurred every half hour of the test. The mass of the specimens was measured every half hour of the test using a AS 60/220 R2 (RADWAG, Poznan, Poland) analytical balance with a measurement accuracy of ±0.01 mg. Changes in the outer diameter of the specimens were measured using a digital micrometer with an accuracy of 0.001 mm. All tests were conducted for 2 h under dry friction conditions without the addition of lubricant.

Three wear parameters were determined: mass wear intensity factor *I_mw_*, relative mass loss Δ*m*/*m*_0,_ and linear wear intensity factor *I_lw_*.

The mass wear intensity factor *I_mw_* was calculated as the mass change Δ*m* per friction surface *S* and unit of friction time *t*, according to the following formula:

(1) $$I_{mw}=\frac{∆m}{S\cdot t}$$

where Δ*m*—mass loss (mg), *S*—friction surface (cm^2^), and *t*—friction time (h).

The relative mass loss Δ*m*/*m_i_* was calculated as a mass change Δ*m* in relation to the initial mass of the specimen *m*_0_:

(2) $$\frac{∆m}{m_{0}}=\frac{m_{0}-m_{f}}{m_{0}}$$

where Δ*m*—mass loss (mg), *m*_0_—initial mass of specimen (mg), and *m_f_*—final mass of specimen (mg).

The third parameter was the linear wear intensity factor *I_lw_* of the specimen expressed as a reduction in thickness of the alloyed zone according to the following formula:

(3) $$I_{lw}=\frac{∆{th}_{AZ}}{t}$$

where Δ*th_AZ_*—reduction in the thickness of the alloyed zone measured as the change in the dimension of the outer diameter of the specimen divided by 2 (µm); *t*—friction time (h).

### 2.4. Temperature Distribution Across the Axis of the Laser Track

In the case of the production of WC-Al composite layers, a very important parameter is the temperature reached in the molten pool during the laser action. The idea behind the formation of composite layers is to preserve the high hardness and wear resistance of the strengthening particles, in this case, WC; so, during the re-melting process, the particles must not be completely melted. The mathematical model proposed by Ashby and Esterling [30,31,32] was adapted to determine the temperature profile across the laser track axis of the WC-Al composite layers.

The mathematical model proposed in this study, like any model, has to be based on specific assumptions. The temperature reached during laser processing depends on many factors, both process and material. As with any model, steps were taken at the outset to simplify the model in order to simplify the calculations and also to make it more useful. According to the source data [30,31,32], preliminary assumptions were formulated to simplify the use of the model. Thus, in terms of materials, the following was assumed:

- The absorptivity of the outer surface of specimen *A* is a constant value and independent of temperature;
- The physical properties of the alloyed and alloying materials are constant and independent of temperature.
- The following assumptions were formulated with regard to process parameters:
- The initial temperature *T*_0_ is equal to 293 K and is invariant during laser processing;
- The laser beam moves in the x-axis direction at a constant scanning speed *v_l_*;
- The thickness of the alloyed zone *th_AZ_* is defined as the intersection point of the temperature distribution profile and the melting point of the substrate material (913 K) that will be reached.

According to the method proposed by Ashby and Esterling, the temperature distribution was determined for the axis of a laser track characterized by a laser beam with a Gaussian energy distribution. This model assumes that a laser beam with a power *P* and radius *r_B_* moves in the scanning direction *x* with scanning rate *v_l_*. Such movement of the laser beam causes increase in the temperature *T(z,t)* at each point below the center of the laser track for *y* = 0 according to the following formula:

(4) $$T\left(z,\;t\right)=T_{0}+\frac{A\cdot P}{2\cdot \pi \cdot \lambda {\cdot v}_{l}{\cdot \left[t\cdot \left(t+t_{0}\right)\right]}^{\frac{1}{2}}}\times exp\left\{-\left(\frac{{\left(z+z_{0}\right)}^{2}}{4\cdot \alpha \cdot t}\right)\right\}$$

where *T(z,t)*—temperature in the axis of the laser track at the depth *z* (K); *T*_0_—initial (ambient) temperature (K); *A*—absorptivity of the specimen surface; *P*—power of the laser beam (W); λ—thermal conductivity of material (W·m^−1^·K^−1^); *v_l_*—scanning rate of laser beam (m·s^−1^); *t*—interaction time (s); *t*_0_—time constant (s); *z*—depth in the axis of the laser track below the top-surface (m); *z*_0_—depth constant (m); *α*—thermal diffusivity of the material (m^2^·s^−1^).

The thermal diffusivity *α* of a material is defined as the quotient of its density *ρ* and specific heat *C_p_* multiplied by its thermal conductivity *λ*, according to the following equation:

(5) $$\alpha =\frac{\lambda }{\rho {\cdot C}_{p}}$$

where λ—thermal conductivity of material (W·m^−1^·K^−1^); ρ—density of the material (kg·m^3^); *C_p_*—specific heat of the material (J·kg^−1^·K^−1^).

The thermal cycle of the laser treatment is characterized by two constants: time *t*_0_ and depth *z*_0_. The first one is defined as the time required for heat diffusion over a distance equal to the laser beam radius *r_B_*:

(6) $$t_{0}=\frac{r_{B}^{2}}{4\cdot \alpha }$$

where *r_B_*—radius of laser beam (m); *α*—thermal diffusivity of the material (m^2^·s^−1^).

The depth constant *z*_0_ is related to the distance from the top surface where heat could diffuse during the laser beam action in terms of the interaction time *t*. The interaction time is defined as the quotient of the laser beam radius to the scanning rate *t = r_B_*/*v_l_*.

When *t* >>*t*_0_, the depth constant *z*_0_ can be determined using the following formula:

(7) $$z_{0}^{2}=\left[\frac{r_{B}}{e}\cdot {\left(\frac{\pi \cdot \alpha \cdot r_{B}}{v_{l}}\right)}^{1/2}\right]$$

where *r_B_*—radius of laser beam (m); *e*—base of natural logarithms; *α*—thermal diffusivity of the material (m^2^·s^−1^); *v_l_*—scanning rate of laser beam (m·s^−1^).

When *t* << *t*_0_, the depth constant *z*_0_ can be calculated using the following equation:

(8) $$z_{0}^{2}=\frac{\pi \cdot \alpha \cdot r_{B}}{2\cdot e\cdot v_{l}}$$

where *α*—thermal diffusivity of the material (m^2^·s^−1^); *r_B_*—radius of laser beam (m); *e*—base of natural logarithms; *v_l_*—scanning rate of laser beam (m·s^−1^).

The physical properties of the materials considered in the temperature calculations are presented in Table 1. The averaged values of the physical properties of tungsten carbide and aluminum, 0.7 WC + 0.3 Al, were considered for calculations.

## 3. Results and Discussion

**Figure 3:**
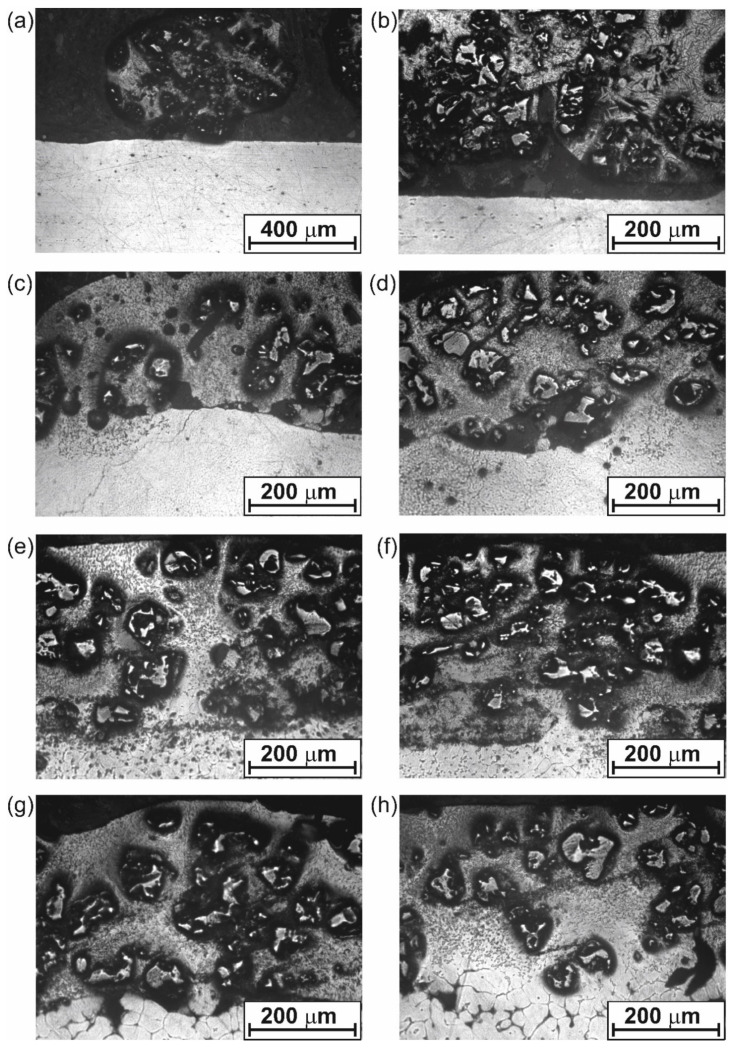
OM images of the microstructure of 7075 alloy after laser surface alloying with WC-Al using laser beam power of (**a**,**b**) 0.65 kW, (**c**,**d**) 0.91 kW, (**e**,**f**) 1.17 kW, (**g**,**h**) 1.3 kW.

Figure 3 presents the microstructure of the WC-Al composite layers produced on the 7075 alloy using different laser beam powers. All layers contained unmelted WC particles in an aluminum-based matrix. The power P used during processing strongly affected the thickness of the alloyed zone (WC-Al composite layer), as well as the percentage of the WC particles. The lowest average thickness of the WC-Al composite layer (198 ± 36 µm) was characteristic of the lowest applied laser beam power (0.65 kW). The increase in laser beam power was the reason for the increase in thickness of the alloyed zone *th_AZ_*. However, an increase in laser beam power from 1.17 kW (Figure 3e,f) to 1.3 kW (Figure 3g,h) resulted in a slight increase in thickness, from 360 ± 19 µm to 387 ± 21 µm, respectively.

The calculated average thicknesses of the alloyed zone deviated from those observed in the microstructure image. For example, the layer formed using *P* = 0.65 kW was characterized by an average thickness of 198 µm, while the OM image of its microstructure shown in Figure 3a suggests a thickness of twice that amount. The differences resulted from the methodology of measuring the thickness of the alloyed zone. Measurements were taken at equal intervals on the microstructure image, resulting in a value of 0 in areas where the layer was not formed.

The laser beam power also affected the percentage of re-melted substrate material in the composite layer. This can be easily determined from the relationship between the thickness of the pre-placed alloying paste *th_paste_* and the thickness of the alloyed zone produced *th_AZ_*. The percentage of substrate material in the WC-Al composite layers is summarized in Table 2. It was obvious that the increase in laser beam power caused an increase in the thickness of the alloy zone *th_AZ_*. This phenomenon was caused by the increased power density of the laser beam, which generated a higher temperature on the surface of the workpiece and, simultaneously, caused interaction with the material at greater distances from the surface. As a result, as the power of the laser beam increases, the depth to which the laser beam interacts with the substrate material also increases. As a result, the alloyed zone produced contains not only material from the melted alloying paste but also some substrate material heated above the melting point. If the depth of the alloyed zone *th_AZ_* increases, the proportion of substrate material in the alloyed zone increases simultaneously. Hence, the determined average thicknesses of the alloyed zone *th_AZ_* should be given together with the percentage of the substrate material in this zone. The role of the substrate material, which has become an integral part of the molten pool as a result of exceeding the melting point, is important. At a constant value of paste thickness *th_paste_* = 160 µm, an increase in the thickness of the alloyed zone *th_AZ_* results in an increase in the dilution ratio *DR* of the alloying material, which can be easily calculated from the following equation [33]:

(9) $$DR=\frac{{th}_{paste}}{{th}_{AZ}}$$

where *DR*—dilution ratio; *th_paste_*—thickness of pre-coated paste with alloying material; *th_AZ_*—thickness of produced laser surface alloyed zone.

The amount and redistribution of alloying material in the alloyed zone is related not only to the laser processing parameters but also to the thermal properties of the materials used and convection in the molten pool [33,34]. The proportion of alloying material in the molten pool can be determined by the dilution ratio factor (*DR*). A high *DR* value suggests a large amount of alloying elements in the alloyed zone.

The calculated *DR* values are presented in Table 2. Based on them, it can be concluded that, the higher the laser beam power, the lower the dilution ratio factor. Simultaneously, the decrease in *DR* is accompanied by an increase in the percentage of the substrate material in the alloyed zone. This means that the increasing thickness of the alloyed zone, *th_AZ_*_,_ caused by increased laser beam power, is accompanied by a decreasing percentage of the alloying material in the alloyed zone. Consequently, with the increase in the laser beam power, the percentage of WC particles in the WC-Al composite layers decreases. As can be seen in Figure 3a,b, the percentage of tungsten carbide particles in the microstructure of the layer is highest for the layer formed with the lowest laser beam power. The higher *P* value is the reason for the increasing thickness of *th_AZ_* and, at the same time, the decreasing percentage of tungsten carbides in the composite layer (Figure 3c–h). The role of WC particles in the WC-Al composite layers is to increase wear resistance; hence, their contribution will be fundamental in interpreting the results of wear resistance tests.

The WC-Al composite layer formed with a laser treatment parameter of *P* = 0.65 kW showed unsatisfactory adhesion to the substrate, as can be clearly seen in Figure 3a,b. This layer was characterized by high thickness inhomogeneity and high discontinuity. In some areas, the layer has the form of a bullet-shaped growth that looks like a solidified drop (Figure 3a). In the area around this drop, the layer did not form at all. Some signs of discontinuity in the layer, as well as poor adhesion to the substrate material, were also observed in some areas of the WC-Al layer formed at a laser beam power of 0.91 kW (Figure 3d). The other composite layers produced were well adhered to the substrate material (7075 alloy).

**Figure 4:**
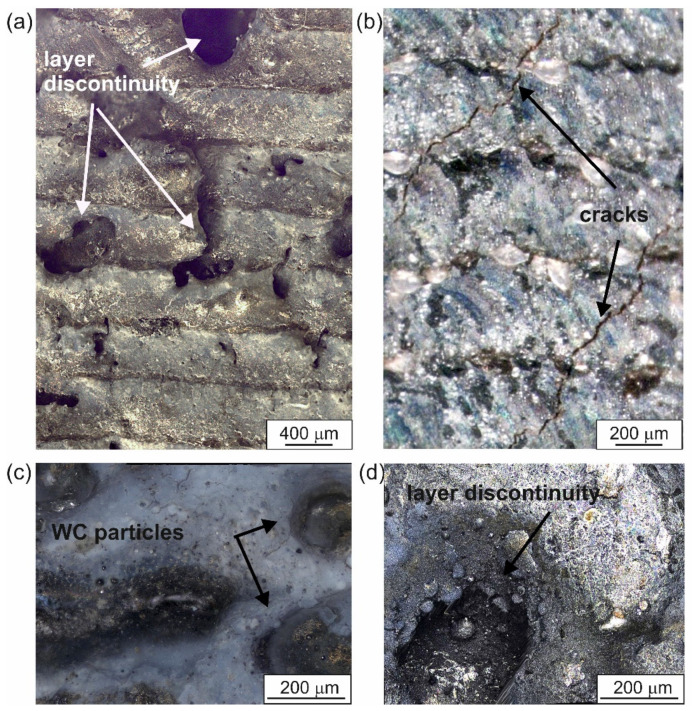
The surface morphology of the specimen laser-alloyed with a laser beam power of 0.65 kW recorded by the digital microscope: (**a**) image of several adjacent laser tracks, (**b**–**d**) details of surface topography.

**Figure 5:**
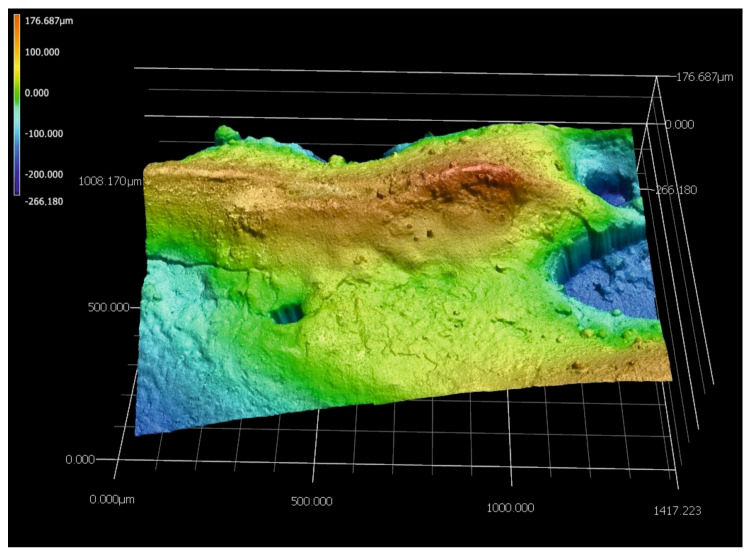
The 3D image of the surface topography of the specimen laser-alloyed with a laser beam power of 0.65 kW recorded by the digital microscope.

**Figure 6:**
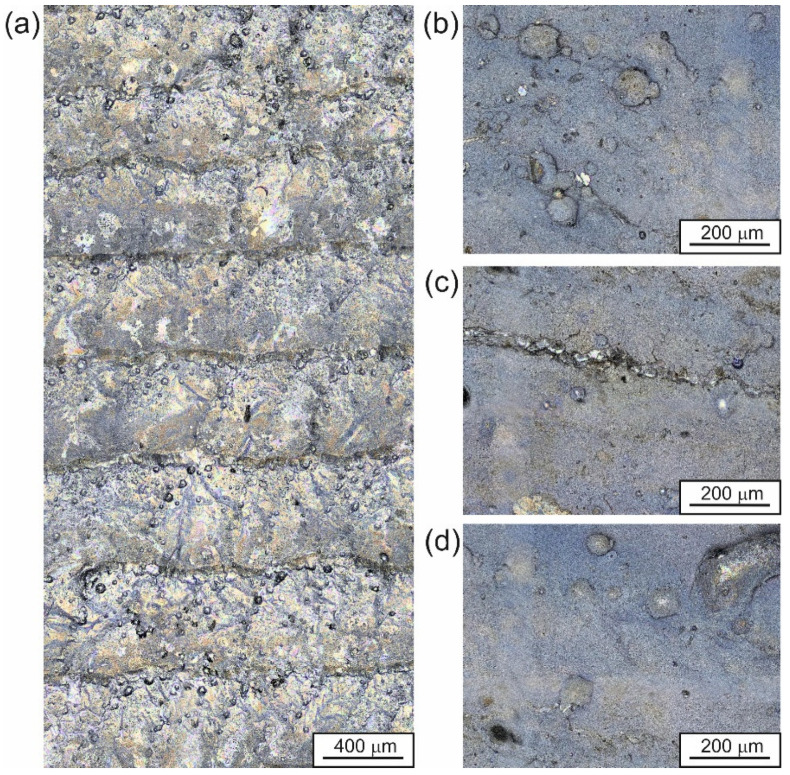
The surface morphology of the specimen laser-alloyed with a laser beam power of 1.17 kW recorded by the digital microscope: (**a**) image of several adjacent laser tracks, (**b**–**d**) details of surface topography.

**Figure 7:**
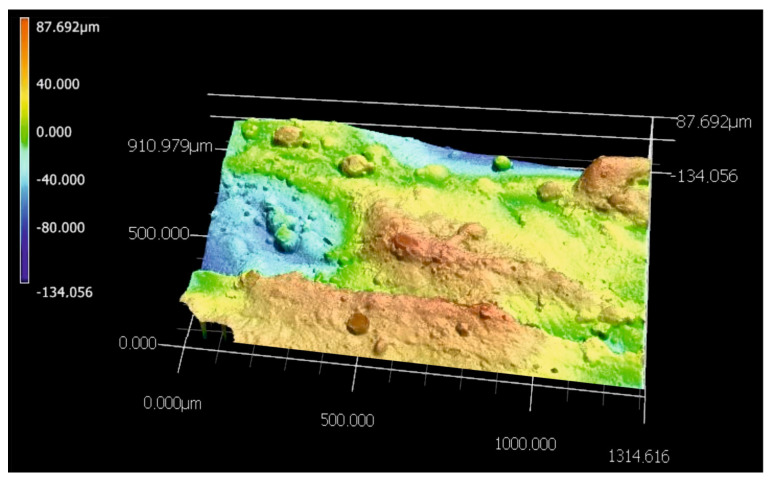
The 3D image of the surface topography of the specimen laser-alloyed with a laser beam power of 1.17 kW recorded by the digital microscope.

The quality of the produced WC-Al composite layers was also investigated based on the surface topography observations. Two specimens were observed: a laser alloyed layer produced at a laser beam power of 0.65 kW (Figure 4 and Figure 5), which represents unsatisfactory adhesion, as well as a specimen treated at a laser beam power of 1.17 kW (Figure 6 and Figure 7) corresponding to good adhesion.

The layer produced using the lowest *P* values showed many cracks (Figure 4b). In addition, discontinuities (Figure 4a,d) were detected in some areas. A 3D image of the surface topography of the specimen modified with a laser beam of *P* = 0.65 kW was presented in Figure 5. Obvious signs of discontinuity and porosity of the layer were clearly visible. The surface of the composite layer is so heterogeneous that the difference between the highest and lowest measurement points was as much as 442 µm. This value is more than twice the average depth of the alloyed zone *th_AZ_*, indicating that the layer is highly discontinuous and heterogeneous.

A different surface topography was represented by a WC-Al layer formed on the 7075 alloy using *P* = 1.17 kW (Figure 6 and Figure 7). In this case, the layer was more uniform with respect to surface topography. No cracks or pores were observed (Figure 6). Considering the 3D image of the surface (Figure 7), it was noted that the difference between the lowest and highest measurement points was 221 µm. This value was two times lower than that measured for the layer produced at a laser beam power of 0.65 kW.

**Figure 8:**
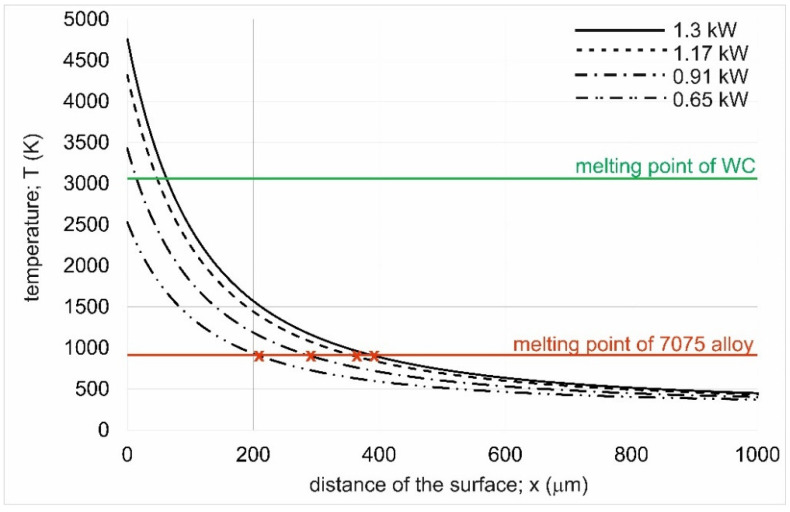
Temperature distribution profiles of the 7075 alloy laser-alloyed at a laser beam power of 0.65 kW, 0.91 kW, 1.17 kW, and 1.3 kW.

It is obvious that the laser beam power affects the phenomena occurring during laser processing. In the case of the present study, the power of the laser beam was selected such that aluminum melting occurred without complete melting of the WC particles. Only such an assumption could produce composite layers strengthened with tungsten carbide particles. However, for the lowest laser beam power, unsatisfactory results were obtained in terms of layer adhesion and quality. The cause of this situation could be the temperature distribution over the cross-section of the laser-treated material. Therefore, the formulas proposed by Ashby and Esterling [30,31,32] were adapted, and the temperature distribution profile was determined during laser melting with beam powers of 0.65 kW, 0.91 kW, 1.17 kW, and 1.3 kW. The results are compiled in Figure 8.

It was found that the WC melting point was reached for the layers produced at laser beam powers of 0.91 kW, 1.17 kW, and 1.3 kW. Only the WC-Al composite layer produced using *P* = 0.65 kW did not reach the melting point of tungsten carbide. It is noteworthy, however, that each of the temperature distribution profiles shows a decrease in temperature as the distance from the treated surface increases. It is clear that even if the surface melting point of tungsten carbide was reached, the WC particles were not fully melted due to the intense convective and gravitational movements in the molten pool. To fully melt the WC particles, a temperature higher than its melting point would have to be reached at a depth equal to or exceeding the thickness of the pre-coated paste with alloying material. The fact that, for a laser beam power of 0.65 kW, the maximum temperature in the laser track axis was 2530 K clearly indicates that there was no melting of the WC particles, which may significantly affect the solidification process of the molten pool. First, the lowest laser beam power was the reason for melting only a small depth of substrate material (Table 2), which means a very high proportion of tungsten carbide present in the produced layer. Since there was no melting or over-melting of the strengthening phase during the solidification of the molten pool, there may have been a problem with the formation of the composite layer involving insufficient wettability of the WC phase with liquid aluminum [23]. Such a problem was described in the papers [20,21,23] regarding the composite layers produced using the laser technique. Low wettability could have been the reason for the formation of cracks in the layer produced at the laser beam power of 0.65 kW. For metal–ceramic composites, the wettability of the reinforcing particles through the metallic matrix is an important factor affecting the strength of the bond between the particles and the matrix. In general, the lower the value of the wetting angle of the ceramic particles by the liquid matrix metal, the better the bond strength between them. In the case of WC particles, the highest wettability is obtained for metals such as iron, cobalt, and nickel [23]. However, in the case of the tungsten carbide-aluminum system, the wetting angle is 135° [23]. Such a value of the wetting angle makes it impossible to achieve a strong bond between the reinforcing particles and the metallic matrix. In such situations, the best solution is to add elements to the metallic matrix that increase wettability, such as silicon, or to induce a reaction between the particles and the matrix due to superfusion of the ceramic particles [23,35]. Over-melting of the ceramic particles at the particle/matrix interface causes the formation of new phases in this area, resulting in the diffusion bonding of the WC particles to the metallic matrix. For laser manufacturing processes of ceramic/metal composite layers, it is, therefore, important to achieve a temperature compromise that will ensure both the formation of a liquid metallic matrix and the over-melting, rather than complete melting, of the ceramic particles. For this reason, it is important to determine the temperature profile in the molten pool, which indicates whether the WC-Al system under consideration will result in over-melting of the WC particles but without complete melting. It is clearly evident, in Figure 7, that only in the case of the specimen alloyed with a laser beam power of 0.65 kW was the melting point of the tungsten carbide not reached. Even the maximum temperature generated on the top surface of the processed sample (2530 K) is much lower than the WC melting point (3058 K). This situation is the reason for the lack of a stable diffusion bond at the interface between the tungsten carbide particles and the aluminum matrix.

Another reason for the poor quality and insufficient adhesion of this layer to the substrate material may be the melting of only the alloying paste without melting the substrate material, achieving a high dilution ratio (*DR* of nearly 100). The temperature distribution shown in Figure 8, as well as the theoretical values of *th_AZ_*, are purely theoretical and based on a number of simplifications; hence, it may be that the substrate material was not remelted at all under experimental conditions. The theoretical thickness of the layer formed at *P* = 0.65 kW was 205 µm (red point x marked at the intersection of the melting point of the 7075 alloy and the temperature distribution profile). However, in reality, due to some simplifications of the mathematical model, this value may have been less, even less than the thickness of the pre-coated paste *th_paste_*. If that were the case, then, actually, at the molten pool/solid substrate material boundary, they might not bond well.

The Ashby and Esterling model is useful for predicting the thickness of layers produced by laser alloying. By using the model, long and tedious preliminary studies focused on the selection of laser treatment parameters can be omitted. This is particularly important when the layers to be produced are to be composite in nature, and the reinforcing particles, as in this study, are not to be completely melted by the laser beam. For this reason, a preliminary study consisting of the theoretical selection of laser processing parameters was first carried out before starting the experiments. For this purpose, a mathematical model prepared by Ashby and Esterling was used, on the basis of which laser beam power values generating temperatures that were too low, as well as those that were too high, were rejected. Too low a temperature and an unfavorable temperature distribution in the material would result in too high a dilution ratio and poor bonding of the layer to the substrate. In addition, unmelted WC particles would have insufficient bonding to the metallic matrix of the layer. In contrast, too high *P* values would result in too high a contribution of the substrate material, as expressed by the value of *th_subs_*, and too low a value of the *DR* parameter. This means that too high a laser beam power results in a composite layer with a low percentage of the strengthening phase (WC particles). The validity of using the Ashby and Esterling model to predict the depth of the alloyed layer is confirmed by a comparison of theoretical and experimental thicknesses of WC-Al composite layers (Table 3). In all cases considered, a high correspondence was obtained between the theoretical and experimental thickness of the alloyed zone. Obviously, the obtained differences in *th_AZ_* values are due to some simplifications of the model described in the experimental methodology. Hence, it is impossible to obtain full agreement between theory and experiment.

**Figure 9:**
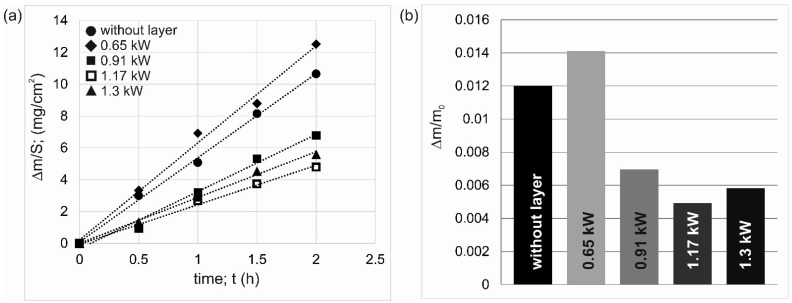
Results of wear resistance tests: (**a**) mass loss of specimens on a unit of friction surface vs. time of friction, (**b**) relative mass loss of specimens.

**Figure 10:**
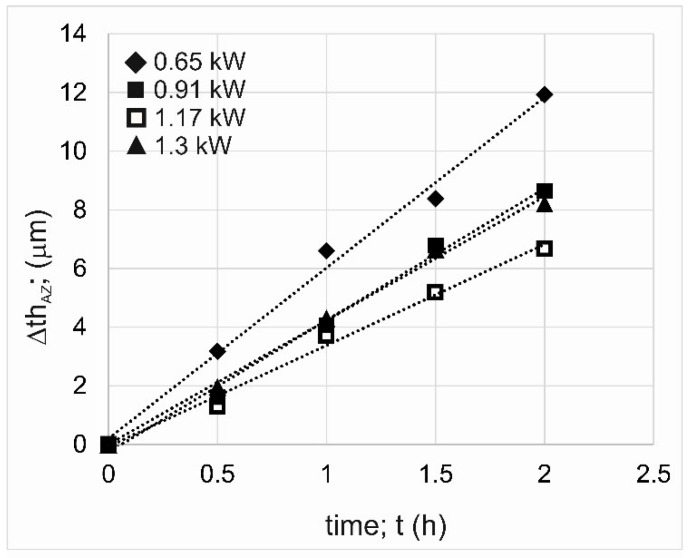
Results of wear resistance tests: reduction in the thickness of the alloyed zone *th_AZ_*.

The tribological properties of laser-alloyed specimens were analyzed based on three characteristics: the mass wear intensity factor *I_mw_* (Figure 9a), the relative mass loss Δ*m*/*m_i_* (Figure 9b), and the linear wear intensity factor *I_lw_* (Figure 10). The results in quantitative form are also shown in Table 4. Considering the mass wear intensity factor, it should be noted that the layer formed using a laser beam power of 0.65 kW was characterized by a higher value of *I_mw_* than alloy 7075 without a layer, with values of 6.094 mg/cm^2^/h and 5.288 mg/cm^2^/h, respectively. Significantly lower *I_mw_* values were determined for the other layers (Table 4).

The highest wear resistance was represented by the layer produced at a laser beam power of 1.17 kW (*I_mw_* = 2.475 mg/cm^2^/h). The same information was provided by the results of the wear test presented in the form of relative mass loss (Figure 9b). The highest Δ*m*/*m_i_* ratio (0.01411) was characteristic of the layer produced at the laser beam power of 0.65 kW. The lowest Δ*m*/*m_i_* ratio (0.00497) was characteristic of the layer produced at the laser beam power of 1.17 kW.

Very informative parameters describing the tribological characteristics of the specimens are the linear wear intensity factor *I_lw_* and the reduction in thickness of the WC-Al composite layer Δ*th_AZ_*. As in the case of indices based on mass loss, the layer formed using *P* = 0.65 kW showed the highest linear wear (Figure 10). In this case, as a result of the two-hour wear test, the thickness of the WC-Al composite layer was reduced by a value of 11.93 µm. The highest tribological properties were demonstrated by a specimen laser-alloyed using a laser beam power of 1.17 kW, for which the measured WC-Al layer thickness reduction was 6.66 µm.

It was expected that an increase in laser beam power would be accompanied by reduced wear resistance due to a diminished percentage of WC particles and a significantly increased percentage of re-melted substrate material in the produced layer. Such a situation was described in the case of metal matrix composite layers strengthened with tungsten carbide particles formed on an Inconel 600 alloy [29]. However, in the case of the present study, the layer produced at the lowest laser beam power showed the lowest wear resistance, regardless of whether wear parameters relating to mass loss or layer thickness reduction were considered. The reason for this situation was insufficient adhesion of the layer to the substrate material, as well as a problem with the wettability of WC particles by liquid aluminum during the laser beam action. As a result, the tungsten carbide particles were not well bonded to the metallic matrix, which caused them to peel out of the matrix during the wear test. Free WC particles were getting between the tested specimen and the counter-specimen during the wear test and working as an abrasive. The increased mass loss of the specimen laser treated with *P* = 0.65 kW was due to two factors. The first was the peeling of WC particles from the metallic matrix, which in itself reduced the mass of the specimen intensively. The second reason was the interaction of free WC particles as an abrasive, which accelerated the wear of this specimen.

**Figure 11:**
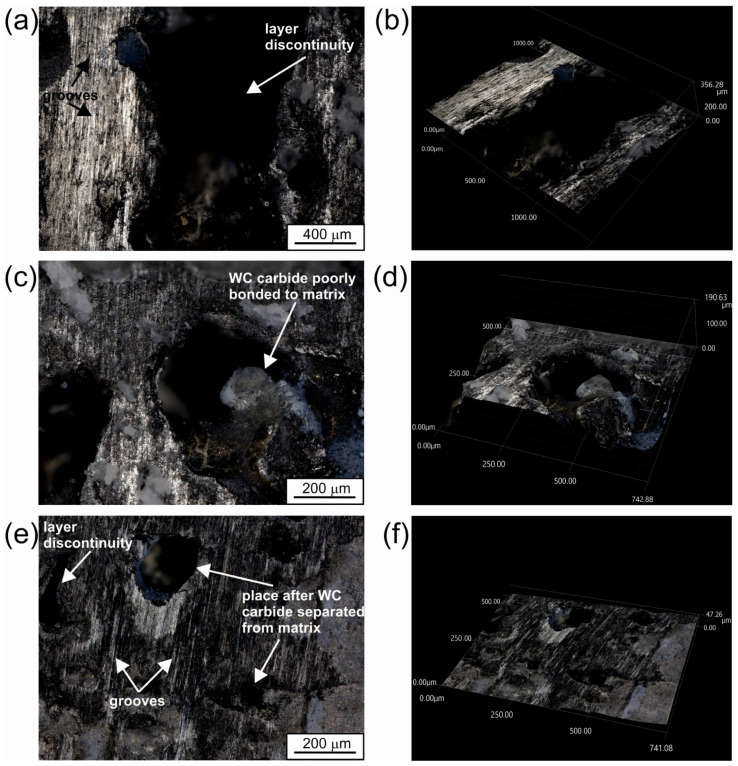
The worn surface of the specimen laser alloyed using a laser beam power of 0.65 kW: (**a**,**c**,**e**) 2D images; (**b**,**d**,**f**) 3D images.

To prove the theory of poor bonding of WC particles to the metallic matrix of the layer produced with the lowest laser beam power, the specimen surface was observed after the wear test. Images registered with the digital microscope are shown in Figure 10. For better visualization of the surface topography, 2D and 3D images were recorded. First, the presence of discontinuities in the layer was confirmed (Figure 11a,b), which was also visualized using an optical microscope by analyzing the cross-sectional view of the layer (Figure 3a). The primary wear mechanism clearly visible in Figure 11a,c is abrasive wear, the presence of which is confirmed by numerous grooves. In some areas of the worn surface (Figure 11e), porosities can be seen, which were formed due to the separation of the WC particle from the metallic matrix. These areas are visible as holes in the sample surface, the topography of which can be seen very clearly in Figure 11f. The most convincing evidence for the theory of poor bonding of WC particles to the aluminum matrix in the layer produced with a laser beam power of 0.65 kW is Figure 11c,d. A hole in the friction surface is also visible in this area, but, here, a WC carbide is still visible inside it. Analyzing the 3D image of this area (Figure 11d), it can be seen that this particle is lying freely in the hole and there is actually no bond, either chemical or adhesive, between it and the metallic matrix. This carbide was exposed as a result of the abrasion of the aluminum matrix and would probably have separated from the surface during a further wear test. Thus, based on Figure 11, it was found that the WC-Al composite layer produced with the lowest laser beam power did not achieve a good bond between the reinforcing WC particles and the aluminum matrix.

**Figure 12:**
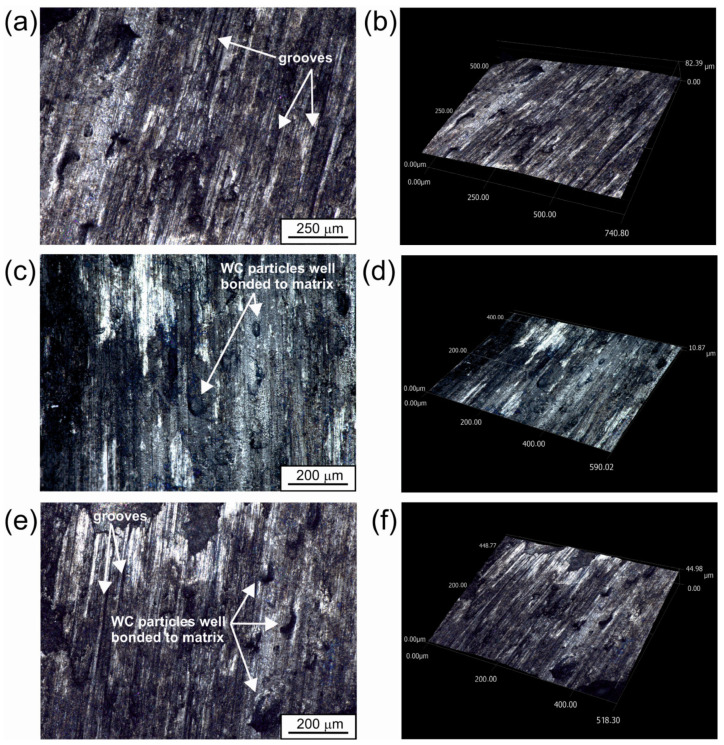
The worn surface of the specimen laser alloyed using a laser beam power of 1.17 kW: (**a**,**c**,**e**) 2D images; (**b**,**d**,**f**) 3D images.

In order to verify that, for the sample characterized by the highest wear resistance, the bond between the WC particles and the matrix is sufficient, 2D and 3D images recorded after the wear test were also analyzed (Figure 12). The composite layer produced with *P* = 1.17 kW after the wear test shows the presence of numerous grooves (Figure 12a,e), indicating an abrasive wear mechanism. The observed worn surface shows no defects in the form of discontinuities or porosity. In addition, no holes indicating separation of WC particles from the matrix were observed. In contrast to the worn surface of the specimen shown in Figure 11, some areas (Figure 12c,e) show protrusions that indicate the presence of WC particles. Analysis of 3D images of these areas (Figure 12d,f) confirms that they are elements that protrude slightly above the friction surface, which may indeed support the theory of good bonding between the tungsten carbide particles and the aluminum matrix in this layer.

In conclusion, it can be found that the WC-Al composite layer formed at a laser beam power of 1.17 kW showed the highest wear resistance, both considering the mass and linear loss of the specimen. The layer showed good quality and satisfactory adhesion to the substrate material. Simultaneously, the worn surface (Figure 12) clearly indicates the presence of tungsten carbide particles well bonded to the aluminum matrix in the layer.

However, it should be noted that the specimen melted with a laser beam power of 1.3 kW showed a slightly lower wear resistance in comparison to the specimen treated using *P* = 1.17 kW. Such an effect is consistent with the general knowledge of the decreasing percentage of reinforcing particles in ceramic–metal composite layers. Such an effect was described in the work [29] describing the influence of laser beam power on the wear resistance of laser-alloyed WC layers produced on Inconel 600 alloy. Generally, the higher the power of the laser beam used to produce the composite layers, the lower the percentage of reinforcing ceramic particles. It was found that increasing the laser beam power from 1.3 kW to 1.82 kW resulted in a more than two-fold reduction in the percentage of reinforcing WC particles in the layer. This was also the reason for a three-fold decrease in wear resistance of the layer produced with a laser beam power of 1.82 kW compared to that produced with a laser beam power of 1.3 kW [24]. Therefore, the lower wear resistance demonstrated by the WC-Al composite layer produced with a laser power of 1.3 kW compared to that formed with a laser beam power of 1.17 kW is consistent with the generally known trend concerning the influence of the laser beam power on the percentage of reinforcing particles in metal–ceramic composite layers.

The above information would indicate that the layer produced with a laser beam power of 0.91 kW should exhibit higher wear resistance than the layer produced with a laser beam power of 1.17 kW. However, the obtained results indicate the opposite. The reason for the diminished wear resistance of the layer produced using *P =* 0.91 kW in comparison to that produced at *P =* 1.17 kW was the presence of some discontinuities in it (Figure 3d). Moreover, the temperature distribution across this layer indicated that it could be too low to melt the edges of all the WC particles, and, therefore, the bonding between some tungsten carbide particles and the matrix was not fully satisfactory.

## 4. Conclusions

The laser surface alloying technique is appropriate for producing metal matrix composite layers reinforced with ceramic tungsten carbide particles. The quality of the layers and their wear resistance strongly depended on the laser beam power used, as well as the temperature distribution on the laser tracks produced. The main conclusions formulated on the basis of the analyzed results are as follows:

(1) The laser beam power affected the depth of the WC-Al composite layers produced. The lowest thickness of the alloyed zone *th_AZ_* (198 µm) was measured for the layer produced with a laser beam power of 0.65 kW. Increasing the laser beam power to 1.3 kW was accompanied by a twofold increase in the average layer thickness.
(2) The percentage of re-melted substrate material in the molten pool increased with increasing laser beam power.
(3) The laser beam power used during surface alloying strongly affects the bond between the ceramic particles and the metal matrix. In the case of the lowest power of laser beam (0.65 kW), the temperature reached in the molten pool was too low to over-melt the edges of the reinforcing particles. As a result, there was no strong diffusion bond between the particles and the aluminum matrix.
(4) In the case of the WC-Al composite layer produced at a laser beam power of 0.65 kW, its adhesion to the substrate was unsatisfactory. The layer was not well bonded to the substrate material and was characterized by high thickness inhomogeneity and high discontinuity.
(5) The layer formed at the lowest laser beam power was characterized by a wear resistance lower than the 7075 alloy without a surface layer. It was caused by the insufficient adhesion of this layer to the substrate material, as well as a problem with the wettability of WC particles by liquid aluminum during the laser beam action. As a result, tungsten carbide particles were not well bonded to the metallic matrix, which caused them to peel out of the matrix during the wear test. Free WC particles were getting between the tested specimen and the counter-specimen during the wear test and working as an abrasive.
(6) The layer formed using *P* = 1.17 kW showed the highest wear resistance. This layer showed satisfactory quality and adhesion to the substrate material.

## Figures and Tables

**Table 1:** Physical properties of materials used.

	Density *ρ* (kg·m^3^)	Thermal Conductivity *λ* (W·m^−1^·K^−1^)	Specific Heat *C_p_* (J·kg^−1^·K^−1^)	Thermal Diffusivity *α*·(m^2^·s^−1^)	Melting Point *T_m_* (K)
WC	15,700	85.0	280	1.934 × 10^−5^	3058
Al	2700	237	897	9.786 × 10^−5^	933
7075 alloy	2810	134	862	5.532 × 10^−5^	913
0.7 WC + 0.3 Al	11,800	130.6	465.1	2.379 × 10^−5^	-

**Table 2:** The percentage of substrate material in WC-Al composite layers produced at different laser beam powers.

	0.65 kW	0.91 kW	1.17 kW	1.3 kW
*th_paste_* (µm)	160	160	160	160
*th_AZ_* (µm)	198	298	360	387
Percentage of substrate material in layer (%)	19.2	46.3	55.5	58.7
Dilution ratio *DR*	80.2	53.7	44.5	41.3

**Table 3:** The comparison of experimental and theoretical thickness of alloyed zone produced at different laser beam powers.

	0.65 kW	0.91 kW	1.17 kW	1.3 kW
Experimental *th_AZ_* (µm)	198	298	360	387
Theoretical *th_AZ_* (µm)	205	285	355	385

**Table 4:** The results of wear resistance tests for the 7075 alloy without layer and the 7075 alloy after laser alloying using different laser beam powers.

	Without Layer	0.65 kW	0.91 kW	1.17 kW	1.3 kW
*I_mw_* (mg/cm^2^/h)	5.288	6.094	3.593	2.475	2.874
Δ*m*/*m*_0_	0.01201	0.01411	0.00695	0.00497	0.00583
*I_lw_* (µm/h)	-	5.881	3.938	2.528	2.864
Δ*th_AZ_*	-	11.93	8.65	6.66	8.21

## Data Availability

The original contributions presented in this study are included in the article. Further inquiries can be directed to the corresponding author.
